# The Parkinson’s pandemic: prioritizing environmental policy and biological resilience via the gut

**DOI:** 10.1172/JCI205275

**Published:** 2026-03-02

**Authors:** Bianca Palushaj, Robin M. Voigt

**Affiliations:** 1 Department of Neurology and Neurological Sciences, Stanford University School of Medicine, Stanford, California, USA.; 2 Rush Center for Integrated Microbiome and Chronobiology Research,; 3 Department of Internal Medicine, and; 4 Department of Anatomy and Cell Biology, Rush University Medical Center, Chicago, Illinois, USA.

## Introduction

Epidemiological analyses indicate that Parkinson’s disease (PD) incidence has more than doubled in many industrialized regions over the past generation, and projections estimate an over 50% increase globally by 2040. This growth far exceeds what can reasonably be attributed to the aging population, genetics, or improved clinical detection (1). The central question then becomes, why is PD increasing?

PD typically presents in the sixth or seventh decade of life with progressive motor symptoms, including resting tremor, bradykinesia, rigidity, and gait disturbance. However, these clinical markers represent a late-stage manifestation that emerges after approximately 50% of dopaminergic neurons in the substantia nigra have already degenerated (1). The underlying pathology is thought to be the accumulation of misfolded α-synuclein (α-syn) protein into Lewy bodies and Lewy neurites, which disrupt cellular proteostasis, mitochondrial function, and synaptic transmission (1). Crucially, this process begins decades before motor diagnosis: prodromal symptoms such as constipation, anosmia, and REM sleep behavior disorder often precede motor dysfunction by 10–20 years, reflecting early pathology in the gut, olfactory bulb, and brainstem.

The fact that pathology can be observed in the periphery decades before it reaches the brain suggests that we must look beyond the central nervous system to understand the increase in disease burden. Known genetic and molecular pathways explain vulnerability but do not account for why some individuals develop disease while others with identical risk factors do not. More critically, they cannot explain why PD incidence is rising globally at rates that outpace genetic or demographic change. What has changed in the past 30 years to double disease incidence? The answer must lie in environmental exposures and in the biological systems that determine whether susceptible individuals cross the threshold into pathogenesis.

We propose that the answer lies not in the brain but in the gut. While the skin, lungs, and gut all serve as environmental interfaces, the gut is uniquely positioned as the primary site of systemic modulation. Unlike the lipophilic barrier of the skin or the limited biotransformation capacity of the lung, the gut is continuously exposed to a high volume of diverse environmental chemicals — pesticides, solvents, heavy metals, food additives, microplastics, xenobiotics, pathogens, and inflammatory dietary components — over a surface area exceeding 300 m². Crucially, the gut is the only interface hosting a metabolically active microbial ecosystem capable of biotransforming environmental exposures into either neuroprotective or neurotoxic metabolites (2). Even inhaled or dermally absorbed toxins, such as paraquat and trichloroethylene, often converge on the gut through mucociliary clearance, ingestion, or enterohepatic circulation, undergoing microbial metabolism before systemic distribution. Braak staging models propose that α-syn aggregation begins in the enteric nervous system then spreads through the vagus nerve to the central nervous system, which is plausible given the decades-long prodromal phase marked by constipation and gastrointestinal dysfunction (3). The intestinal barrier and microbiome thus serve as the first — and most modifiable — biological filter between the environment and the body.

The thesis of this Viewpoint is straightforward: the gut milieu influences resilience and influences who among genetically or environmentally vulnerable individuals develops PD. If correct, then PD research and clinical strategy must expand beyond neurocentric frameworks and incorporate gut-based prevention and therapeutic approaches.

## The convergence model: PD as erosion of resilience

PD epidemiology implicates environmental exposures, such as pesticides (paraquat, rotenone), solvents (trichloroethylene), and air pollution (PM_2.5_) in PD pathogenesis (4, 5). Individual exposures are significant yet insufficient to explain the rising incidence of PD on their own, suggesting that PD arises from cumulative environmental pressure (the exposome) that reduces host resilience. We posit that the gut influences resilience through microbial composition and function and barrier integrity. We categorize exposures that disrupt gut resilience into two major groups below.

### Chemical selectors of microbial pathobionts.

**Figure 1:**
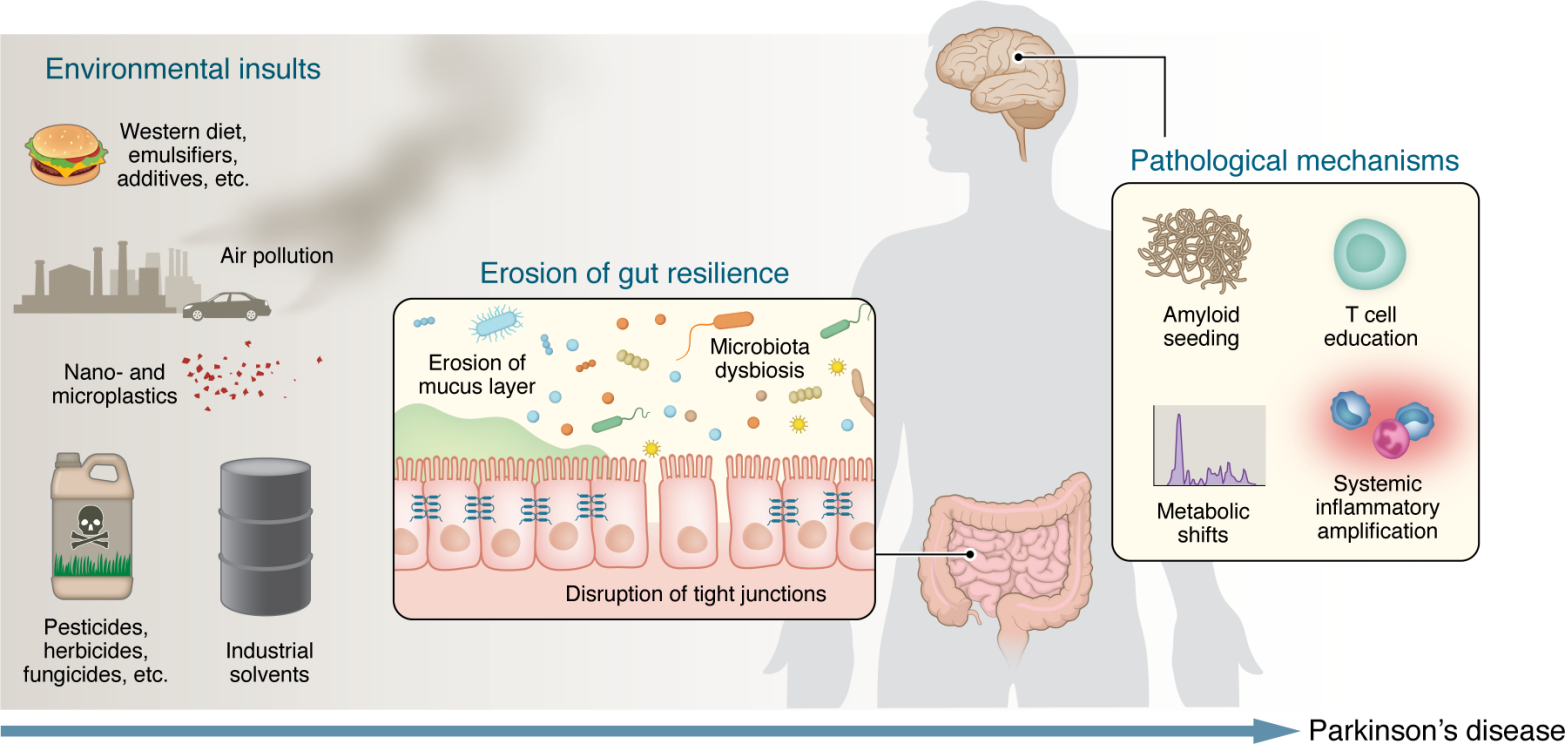
Environmental insults reduce gut resilience and initiate convergent mechanisms that drive Parkinson’s disease. A lifetime of environmental insults (including Western diets and food additives, nano- and microplastics, pesticides and herbicides, industrial solvents, and air pollution) act on the intestinal microbiome and barrier. These exposures promote microbiota dysbiosis, disrupt tight junctions, and erode the mucus layer, collectively reducing gut resilience. Once this peripheral defense is compromised, several mechanistic pathways propagate pathology to the brain: (a) amyloid seeding by bacterial functional amyloids, (b) maladaptive T cell education and autoimmune responses, (c) microbiome-driven metabolic shifts that generate neurotoxic metabolites and reduce short-chain fatty acids, and (d) systemic inflammatory amplification. Together, these processes lower the threshold for α-syn misfolding, neuroinflammation, and neurodegeneration.

Toxins act as selective pressures, favoring taxa with putative detrimental effects. For example, paraquat generates reactive oxygen species that reduce microbial diversity and enrich for *Enterobacteriaceae*, which produce functional amyloids known as curli (6). Similarly, the solvent trichloroethylene and related chemicals inhibit mitochondrial complex I in host tissues while simultaneously restructuring the microbial community to favor sulfate-reducing bacteria like *Desulfovibrio* over neuroprotective, short-chain fatty acid–producing (SCFA-producing) taxa (7, 8). Rodent models exposed to paraquat and trichloroethylene demonstrate that gut dysbiosis precedes motor deficits and nigral neuron loss, suggesting that early microbial shifts may initiate or fuel neuroinflammation (9). Compounded by air pollution, the Western diet, and microplastics that promote gut dysbiosis, these exposures reshape the microbial landscape into a proinflammatory, amyloidogenic, and neurotoxic environment that drives PD biology (Figure 1).

### Barrier disruptors.

Various environmental factors disrupt the intestinal barrier, which consists of the protective mucus layer and tight junction proteins linking epithelial cells. Dietary emulsifiers, such as carboxymethylcellulose and polysorbate-80, erode the mucin layer and disrupt the intestinal barrier in both animals and humans (10). Ingested microplastics and air pollution particles (PM_2.5_) further impair gut barrier function, inducing macrophage activation and inflammation (11). These ubiquitous plastics also concentrate heavy metals and pesticides on their surfaces, effectively ferrying environmental toxins across the intestinal barrier (12). This collective compromise of barrier integrity facilitates the translocation of bacterial endotoxins (e.g., LPS) and environmental toxins into the systemic circulation, promoting inflammation and weakening the blood-brain barrier.

## Mechanisms: how gut vulnerability becomes neurodegeneration

Once the resiliency of the gut is overwhelmed, the consequences ripple to the central nervous system through several interconnected mechanisms.

### Functional amyloids (structural seeding and innate immune activation).

*Enterobacteriaceae* contribute to α-syn pathology by producing CsgA and CsgB, the primary subunits of curli. These β sheet–rich bacterial amyloids are structurally homologous to α-syn, and curli accelerates α-syn aggregation: oral exposure to curli increases α-syn deposition in the midbrain of transgenic models (13). Importantly, curli is also a pathogen-associated molecular pattern (PAMP). By activating the TLR2/MyD88/NF-κB pathways, curli stimulates inflammation and primes microglia toward a hyperreactive state. This dual role as a structural template for α-syn and an inflammatory stimulus makes curli a critical bridge between environmental exposure and neurodegeneration.

### Autoimmunity (T cell education in the gut).

The gut also serves as a training ground for autoimmunity. α-Syn–specific CD4^+^ T cells, present in both the blood and cerebral spinal fluid of individuals with PD, recognize α-syn epitopes and release cytotoxic cytokines (IFN-γ) in the substantia nigra (14). This cascade likely originates in the periphery, where chronic inflammation promotes Th17 and Th1 cell priming (15). Barrier compromise increases antigen sampling, allowing bacterial antigens and α-syn fragments to drive T cell cross-reactivity. Once primed in the gut, these cells possess the capacity to cross the blood-brain barrier and initiate direct cytotoxicity against dopaminergic neurons. In this framework, the gut is the training ground for the activation of an autoimmune attack against the brain.

### Metabolic shunts (kynurenine pathway, hydrogen sulfide, SCFA) and systemic inflammatory amplification.

Dysbiosis further directs metabolism in three neurotoxic directions. The so-called “kynurenine switch” upregulates the enzyme indoleamine 2,3-dioxygenase (IDO1), diverting tryptophan away from the synthesis of neuroprotective serotonin and toward the production of kynurenine-derived neurotoxins like quinolinic acid (NMDA receptor agonist that drives excitotoxicity) and 3-hydroxykynurenine (mitochondrial injury) (16). Simultaneously, sulfate-reducing bacteria (e.g., *Desulfovibrio*) produce pathological excess of hydrogen sulfide (H_2_S), which inhibits cytochrome *c* oxidase (complex IV) inducing mitochondrial impairment and exacerbating α-syn aggregation (8). This microbial output often synergizes with heavy metals (mercury) that can further stimulate the growth of sulfate-reducing bacteria (17). These shifts are compounded by the loss of SCFAs like butyrate, which normally strengthens the intestinal barrier and promotes tolerogenic signaling (7). Consequently, barrier dysfunction permits translocation of bacterial components (LPS, peptidoglycan) into the systemic circulation, engaging TLR2/4 and NLRP3 inflammasomes to release IL-1β and IL-18 (18). These cytokines cross the blood-brain barrier directly to prime microglia toward a hyperreactive state (19). In this primed state, microglia exhibit exaggerated pathological responses to subsequent insults (whether from viral infections, low level environmental toxins, or endogenous α-syn aggregates), converting otherwise subthreshold events into chronic, neuron-damaging inflammation. This mechanism of systemic inflammatory amplification helps explain why dopaminergic neurons undergo degeneration in response to relatively modest stimuli when biological resilience has been compromised.

## A two-pronged therapeutic framework

If PD arises from a lifetime of environmental pressure acting on a vulnerable microbiota-gut-brain axis, then effective intervention requires reducing environmental burden and increasing biological resilience.

### Reduce environmental burden.

We must treat environmental exposures with the same urgency as genetic risk, which will require reforming both environmental and dietary policies. Given the robust epidemiological associations between pesticides/solvents and PD, regulatory action offers the highest-yield preventative strategy. This would include phasing out high-risk agrichemicals (e.g., pesticides, herbicides, and fungicides) and industrial solvents that have strong mechanistic plausibility and consistent observational links in PD (20), as well as promoting regenerative agriculture, which reduces pesticide reliance, increases soil microbiome diversity, and improves food safety. (21). Ultra-processed diets degrade gut resilience: emulsifiers disrupt intestinal barrier function, artificial sweeteners alter microbiome composition, and low-fiber diets reduce SCFA production (22). Reforming diet policies to limit high-risk additives and incentivize consumption of fiber-rich, minimally processed food could yield scalable, population-level benefits.

### Enhance biological resilience.

Environmental exposures cannot be fully eliminated; however, resilience can be strengthened through targeted biological interventions of the gut microbiome. The therapeutic frontier is moving beyond simple probiotics toward precision engineered strains designed to degrade curli amyloids, metabolize xenobiotics, and to secrete protective metabolites (e.g., butyrate, indole derivatives) as well as CRISPR-based strain editing to modulate microbial pathways that interface with α-syn biology (23–25). Barrier restoration and immune modulation strategies include SCFA-producing synbiotics to restore intestinal barrier integrity and regulate immune tone, mucin-supporting oligosaccharides to reinforce the mucus layer, modulators of TLR2/4 signaling to reduce hyperresponsive innate pathways, and small molecules targeting IDO1 or recalibrating kynurenine metabolism (26–28). Given the malleability of the microbiome and intestinal barrier, these approaches offer a tractable route toward disease modification and prevention in high-risk populations.

## Conclusion and call to action

We have spent decades focusing on central mechanisms of PD: dopaminergic neuron vulnerability, mitochondrial dysfunction, α-syn aggregation, and neuroinflammation. These are essential targets, but they largely represent downstream biology. By the time these processes are detectible in the brain, the disease has already passed a critical inflection point. In contrast, converging epidemiologic, experimental, and clinical evidence now places the gastrointestinal tract upstream of this cascade. The gut is where environmental pressure first intersects with host susceptibility. It is where microbial ecology and epithelial barrier integrity determine whether misfolded α-syn seeds arise, whether immune tolerance is lost, and whether systemic immune inflammation primes the brain for degeneration. In this framework, the brain is not the origin of PD pathology but its eventual target.

Crucially, the gut remains therapeutically accessible even after neurologic disease is established. Randomized trials and meta-analyses show that microbiome-targeted strategies — including prebiotics, probiotics, synbiotics, SCFA supplementation, and fecal microbiota transplantation — can improve constipation, motor symptoms, and neuroinflammatory biomarkers in individuals with PD (29). While these interventions are not disease-curing, their consistent demonstration of biological engagement of the microbiota-immune-brain axis raises the possibility that modifying the gut milieu may influence downstream neurodegenerative processes and, potentially, aspects of disease trajectory. Importantly, these approaches are safe, well tolerated, and can be deployed alongside standard dopaminergic therapies. Taken together, the gut represents a target that is both mechanistically upstream and clinically modifiable across the disease course.

If we are serious about reversing global PD trends, we must move upstream. Strengthening peripheral resilience (especially gut resilience) is among the most promising, scalable, and scientifically grounded strategies available. The time has come for PD research and clinical trials to fully engage the microbiota-gut-brain axis. Waiting for pathology to manifest in the brain is a losing strategy. Preventing its initiation in the gut may be the only way to alter the trajectory of this modern epidemic.

## Funding support

This work is the result of NIH funding, in whole or in part, and is subject to the NIH Public Access Policy. Through acceptance of this federal funding, the NIH has been given a right to make the work publicly available in PubMed Central.

- National Institute of Diabetes and Digestive and Kidney Diseases grants U24DK140918, U01DK140921, U01DK140923, U01DK140933, U01DK130936, U01DK140939.

## Version 1 03/02/2026

Electronic publication
